# *SOD1* at the Crossroads: Co-Overexpression of Canonical Antioxidant Response and Noncanonical Hydrogen Sulfide Generation Pathways in Down Syndrome, With Immune Cell Implications

**DOI:** 10.21203/rs.3.rs-8535243/v1

**Published:** 2026-01-13

**Authors:** Karthik Mouli, Anton V. Liopo, Hongbin Wang, Larry J. Suva, Kenneth R. Olson, Paul J. Derry, Thomas A. Kent

**Affiliations:** 1 Center for Genomics and Precision Medicine, Institute of Bioscience and Technology, Texas A&M University Health Science Center, Houston, TX, USA; 2 Department of Chemistry, Rice University, Houston, TX, USA; 3 Center for Biomedical Informatics, Texas A&M University College of Medicine, College Station, TX; 4 Department of Veterinary Physiology and Pharmacology, College of Veterinary Medicine and Biomedical Sciences, Texas A&M University, College Station, TX, USA; 5 Department of Physiology, Indiana University School of Medicine South Bend, South Bend, IN, USA; 6 Department of Biological Sciences, University of Notre Dame, Notre Dame, IN, USA; 7 School of Engineering Medicine, Texas A&M University, Houston, TX, USA; 8 Stanley H. Appel Department of Neurology, Houston Methodist Hospital and Research Institute, Houston, TX 77030, USA; 9 Department of Radiology, Houston Methodist Hospital, Houston, TX 77005 USA

## Abstract

Accelerated immune cell aging is well-recognized feature of Down Syndrome (DS), a condition caused by trisomy of human chromosome 21 (*Hsa21*). DS predisposes individuals to recurrent infections, autoimmunity, low bone mass and leukemia. To investigate potential connections between immune cell dysfunction or disruption in DS, serum transcriptomic and proteomic datasets from DS and euploid individuals were examined. High DS superoxide dismutase 1 (*SOD1*) mRNA expression was consistently found and was strongly associated with an increased odds of inflammatory co-occurring conditions such as pharyngitis. *SOD1* mRNA overexpression was also associated with decreased M2-polarized macrophages, increased resting-memory CD4+ T cells, elevated serum interleukin-16 levels and interferon-γ protein levels, indicative of pathological pro-inflammatory immune dysregulation. *SOD1* mRNA was co-overexpressed with glutathione and thioredoxin-dependent pathways, both are integral to the antioxidative responses and the generation of hydrogen sulfide (H_2_S). Although H_2_S overproduction in DS has been attributed to the overexpression of cystathionine-β-synthase (*CBS*), no consistent *CBS* mRNA elevation was observed in this study. Conversely, the increased expression of a thioredoxin-dependent cysteine catabolism pathway suggests noncanonical H_2_S overproduction in DS distinct from *CBS*. Our findings highlight the unexpected relationship between oxidative stress homeostasis and H_2_S overproduction in DS, extending beyond *Hsa21* trisomy.

## Introduction:

Down Syndrome (DS), the result of trisomy of human chromosome 21 (*Hsa21*) trisomy is the most common chromosomal abnormality in humans^1^. While key characteristics of DS include musculoskeletal anomalies, neurocognitive impairment and congenital heart defects, individuals with DS are also at a higher risk of chronic co-occurring conditions including recurrent respiratory infections, autoimmune diseases, hypothyroidism and transient myeloproliferative disorder^1-7^. Increased lifetime risks of immunological and hematological complications in DS have been associated with the pathological accumulation of intracellular oxidative and metabolic stress ^6-8^.

Oxidative and metabolic stress are well characterized features of DS molecular pathophysiology^9^. The overexpression of several *Hsa21* genes have been linked to impaired mitochondrial function and electron leakage from the mitochondrial inner membrane electron transport chain, resulting in the generation of toxic reactive oxygen species (ROS)^10^. Notably among *Hsa21* resident genes, superoxide dismutase 1 (*SOD1*) - a key component of the cellular response to ROS - is overexpressed in DS and potentially drives ROS generation. SOD1 catalyzes the dismutation of superoxide radical to hydrogen peroxide (H_2_O_2_)^11^, which is normally decomposed to water and diatomic oxygen by catalase and peroxiredoxins. A *SOD1* excess relative to catalase has been observed in DS erythrocytes and was suggested to increase the intracellular accumulation of H_2_O_2_, the precursor to more damaging hydroxyl radicals through reactions with divalent metal ions such as Fe^2+ 6,12-15^. However, the distinct subcellular localizations of *SOD1* and catalase – cytoplasmic and peroxisomal, respectively – suggest the *SOD1*/catalase ratio is insufficient to explain elevated H_2_O_2_ in non-erythrocyte cell types ^16,17^. Moreover, the relationship between the expression of *SOD1* and catalysts of cytosolic H_2_O_2_ decomposition remains to be characterized.

In addition to oxidative stress, DS is also characterized by the increased generation of intracellular hydrogen sulfide (H_2_S), a well-documented and potent inhibitor of mitochondrial respiration ^18-20^. Several studies have linked excess H_2_S generation in DS to to the increased expression of another *Hsa*21 resident gene cystathionine-β-synthase (*CBS*)^21-23^. *CBS* catalyzes the conversion of homocysteine to cystathionine as part of the reverse transsulfuration pathway^21-23^. However, we have previously shown an absence of consistently elevated CBS protein in B lymphocytes from DS individuals^18^.Therefore, it seems likely that other metabolic processes catalyzed by non-*Hsa*21 enzymes contribute to the excess intracellular H_2_S in DS cells.

The increased activity of the enzyme 3-mercaptopyruvate sulfurtransferase (*MPST*) in DS fibroblasts is one such possibility that has been linked to H_2_S overproduction^20,24^. In addition, H_2_S generation though MPST-mediated cysteine catabolism is dependent on the presence of low molecular thiol peptides such as thioredoxins and glutathione, which are integral components of the cellular thiol antioxidant protein axis and whose activity is upregulated in response to oxidative stress^25,26^.

The potential links between ROS and H_2_S metabolism in DS are strengthened by data demonstrating that in addition to an antioxidant role, *SOD1* is also an essential oxidizer of H_2_S ^18,27^. Furthermore, we have shown that the inhibition of *SOD1* activity significantly increased H_2_S levels in DS B lymphocytes ^18^. In the current study, the relationship between drivers of increased oxidative stress including *SOD1* and the transcription of response effectors was investigated to uncover links between ROS and H_2_S metabolism in DS.

## Methods:

### Data access:

Patient white blood cell (WBC) bulk messenger RNA (mRNA) sequencing (RNA-seq) and serum proteomics data were collected by the University of Colorado Linda Crnic Institute’s Human Trisome Project, aligned to the human reference genome using RSEM^28^ and made publicly available by the National Institutes of Health Investigation of Co-occurring conditions across the Lifespan to Understand Down syndromE (INCLUDE) data sharing program (https://portal.includedcc.org)^2,3,29^, supported by National Heart, Lung and Blood Institute grant U2CHL156291. All data analyzed in this report were collected under a protocol approved by the Colorado Multiple Institutional Review Board^2^ and de-identified prior to dissemination. Informed consent was obtained from all subjects and/or their legal guardian(s) prior to sample collection. All methods in this study were performed in accordance with the relevant guidelines and institutional regulations.

### Analysis of bulk RNA-seq data:

Gene mRNA count data from 270 individuals with Down syndrome (DS) and 146 euploid individuals were aggregated in R 4.4.1 using the dplyr library^30^ (version 1.1.4; https://github.com/tidyverse/dplyr). The age range of participants was between 0.5 and 69.1 years, with 210 female and 206 male individuals. Gene counts were TMM-normalized to counts-per-million (CPM), low-expressed genes (CPM < 1.0) excluded from downstream analyses and differential gene expression in DS versus euploid assessed using edgeR (version 4.0) ^31^. P-values were calculated using the edgeR pipeline’s negative binomial model and likelihood ratio test for DS versus euploid mRNA expression and adjusted for multiple comparisons using the Benjamini-Hochberg procedure ^32^.

### Analysis of serum proteomics data:

For individuals with mRNA expression data, serum proteomics data was also available for 260 euploid and 113 DS individuals. Serum proteomics profiles were aggregated, log_2_-normalized and tested for statistical significance between cohorts in R 4.4.1 using the dplyr and stats libraries ^30^.

### Gene set enrichment analysis (GSEA) via active protein interaction subnetworks:

GSEA incorporating protein-protein interaction information was performed on log_2_-transformed mRNA expression fold changes obtained from the edgeR pipeline using the pathfindR library (version 2.4.1)^33^. In brief, protein-protein interaction information was obtained from the Search Tool for the Retrieval of Interacting Genes/Proteins (STRING) database and used to generate subnetworks of biologically related and differentially expressed genes. Subsequently, GSEA was performed on gene subnetworks and results mapped onto Reactome biological pathway gene sets^34^.

### Analysis of clinical conditions:

Clinical diagnosis data from each of the 146 euploid and 270 DS individuals was obtained from the INCLUDE data sharing program and aggregated in R^30^. Cohorts of high and low SOD1-expressing DS individuals were defined as the 1^st^ and 4^th^ quartiles of DS SOD1 expression (n = 68 DS individuals, each), respectively. The prevalence of co-occurring conditions was calculated as the number of incidences of a condition divided by the total number of individuals in each cohort. Odds ratios for each condition in SOD1-high versus SOD1-low DS individuals were calculated as (incidences of condition, SOD1-high / incidences of condition, SOD1-low) / (individuals without condition, SOD1-high / individuals without condition, SOD1-low). The calculated odds ratios were tested for statistical significance in R against a null hypothesis of an odds ratio of 1 using Fisher’s exact tests and resulting P-values were adjusted for multiple comparisons using the Benjamini-Hochberg procedure^32^.

### Immune Cell Subpopulation Inference:

Cohorts of high and low *SQOR*- and *SOD1*-expressing euploid and DS individuals were defined as the 1^st^ (high) and 4^th^ (low) quartiles of *SQOR* and *SOD1* gene expression within each group separately. Bulk mRNA transcriptomics data for individuals in each of the 4 cohorts were deconvoluted using CIBERSORTx (http://cibersortx.stanford.edu) against the default peripheral blood cell signature matrix to obtain inferred immune cell frequencies ^35^.

### Visualization:

All heatmaps were generated and hierarchically clustered using R 4.4.1 and the ComplexHeatmap^36^ library. Visualization of individual gene expression patterns across individuals was achieved using the ggforce library^37^. Linear regression analyses and visualization of gene expression P-values against log_2_-transformed fold changes were generated using the ggplot2 library.

## Results:

**Figure 1:**
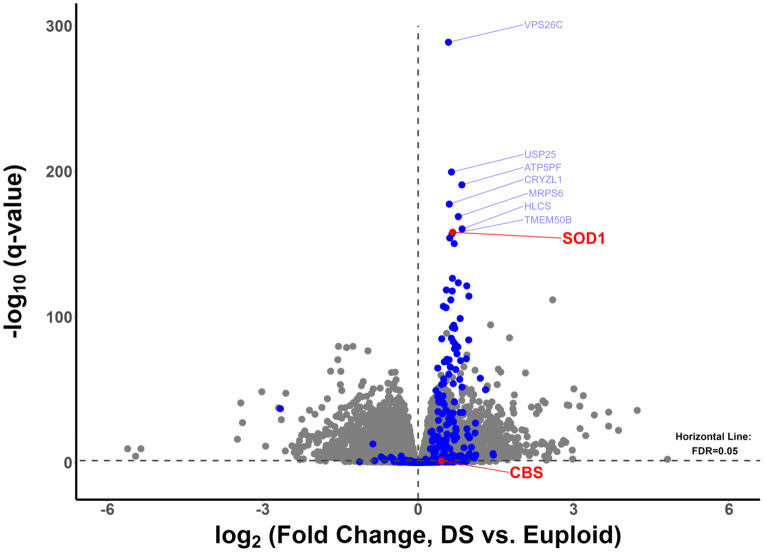
Variability in expression of Hsa21 genes in DS white blood cells relative to euploid, with more consistent superoxide dismutase 1 (*SOD1*) overexpression than cystathionine beta synthase (*CBS*) in DS. Dots represent individual genes, with Hsa21 genes colored in blue. Significant overexpression of *SOD1*, but not *CBS* in Down Syndrome (DS) WBCs relative to euploid individuals. *SOD1* among the most consistently overexpressed genes in DS WBCs (lower q-value). Gene expression fold changes calculated from comparisons between DS and euploid individuals using edgeR. Q-values were calculated from p-values following correction for multiple comparisons using the Benjamini-Hochberg method.

The mRNA expression of most *Hsa21* genes were identified as overexpressed (log_2_(Fold Change, DS vs. euploid) > 0) in DS WBCs relative to euploid, as demonstrated by others ^2,3^ (Figure 1). The mean mRNA log_2_(Fold Change) of significantly overexpressed *Hsa21* genes was 0.626, corresponding to a fold change of 1.54 consistent with triplication of a constitutively expressed locus in *Hsa21* trisomy. *SOD1* was among a cluster of *Hsa21* genes consistently overexpressed in DS individuals versus euploid. Similarly, mRNA overexpression of *Hsa21* genes – *NRIP1*, *SUMO3*, *DYRK1A*, *RCAN1*, *ETS2*, *PKNOX1/PREP1* - previously linked to decreased mitochondrial respiration efficiency and increased ROS generation – was also identified ^38,39^ (Supplementary Figure 1). In contrast, *CBS* mRNA was not significantly overexpressed in DS WBCs (Figure 1), implicating *SOD1* and not *CBS* mRNA overexpression as a salient feature of the DS WBC transcriptome.

**Figure 2:**
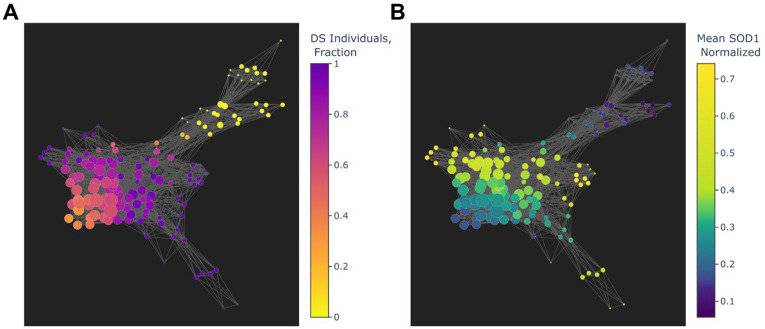
Topological representation of RNA-seq data distinguishes euploid from DS individuals based on Hsa21 gene expression. Graphs generated using an Isolation Forest anomaly score calculated from chromosome 21 gene expression values and Principal Component Analysis as filter functions for the Mapper algorithm. Nodes represent groups of individuals with similar mRNA expression profiles and edges represent similarities between nodes. **A.)** Coloring of nodes by fraction of DS individuals (“1” represents 100% of individuals in a node being from the DS cohort) revealed distinct separation of euploid-predominant nodes from DS based on chromosome 21 gene expression, with notable heterogeneity among DS-dominant nodes. **B.)** Coloring of nodes by mean *SOD1* expression (greater values represent highest mRNA expression) indicated consistently lower *SOD1* expression in euploid-dominant nodes albeit with considerable variation in mean nodal *SOD1* expression across DS-dominant nodes, indicating that variability in *SOD1* expression may contribute to phenotypic variation in DS severity.

To profile the heterogeneity of DS and euploid WBC transcriptomes, topological data analysis was performed to group individuals based on similarities in mRNA expression^40,41^. As a metric of biological dissimilarity due to trisomy, an anomaly score was calculated using the Isolation Forest algorithm^42^ on normalized *Hsa21* mRNA expression values from DS and euploid patient transcriptomics data. *Hsa21* anomaly scores and the first component from Principal Component Analysis (PCA) were used as filter functions for the Mapper algorithm, which was then used to generate graphical representations that grouped individuals with similar transcriptional profiles into nodes^40,43^. Topological data analysis revealed a distinct separation between nodes predominated by DS and euploid individuals, with observed outward flaring of certain DS-dominant nodes suggesting transcriptional heterogeneity within the DS cohort (Figure 2A). While euploid-dominant nodes exhibited consistently lower mean nodal SOD1 expression, we noted considerable variation in *SOD1* expression across DS dominant nodes, suggesting that a potential role for variability in *SOD1* expression across DS individuals contributes to phenotypic variation in symptom severity.

**Figure 3:**
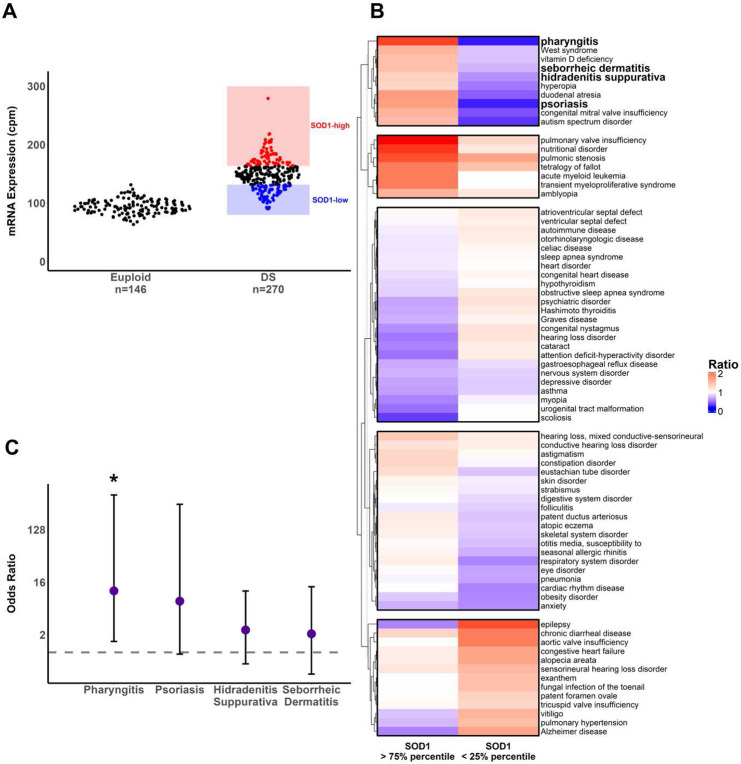
Greater odds of immune dysfunction-associated conditions in DS individuals with higher *SOD1* expression. **A).** Strategy for the identification of highest and lowest *SOD1* mRNA-expressing DS individuals. DS individuals expressing *SOD1* mRNA at levels greater than the 75% percentile of all DS individuals in the dataset were designated as “SOD1-high” (n = 68 individuals), and those expressing *SOD1* lower than the 25% percentile were designated as “SOD1-low” (n = 68 individuals). Y-axis indicates mRNA expression, measured as counts-per-million (cpm) following normalization by edgeR. Dots denote individuals. **B).** Heatmap of prevalences of co-occurring clinical conditions in DS individuals with highest (> 3^rd^ quartile) and lowest (< 1^st^ quartile) *SOD1* mRNA expression, clustered hierarchically using Ward’s method. Color of each cell indicates ratio of prevalence of term in cohort relative to all DS individuals in data set (red: higher, blue: lower prevalence). A notable cluster of diseases were overrepresented in SOD1-high (left column) DS individuals (pharyngitis-autism spectrum disorder) and underrepresented in SOD1-low (right column) DS individuals. Bolded terms indicate diseases of dysregulated immune function within the first cluster, suggesting that increased SOD1 expression is associated with dysfunctional immune responses. **C).** Odds ratios of selected diseases in SOD1-high versus SOD1-low DS individuals. Significantly higher likelihood of pharyngitis in SOD1-high DS individuals versus SOD1-low (Fisher exact test p, adj. < 0.05). Trend toward higher odds ratio with psoriasis, hidradenitis suppurative and seborrheic dermatitis, all diseases of dysregulated immune activity. Dashed line represents odds ratio of 1. Error bars represent 95% confidence intervals of odds ratio. Log-scaled y-axis.

Next, we determined whether *SOD1* mRNA expression in DS individuals was associated with increased frequencies of co-occurring conditions. Cohorts of *SOD1* high- and low-expressing DS individuals were defined as the 1^st^ and 4^th^ quartiles of DS *SOD1* expression (Figure 3A). The ratios of prevalences of co-occurring conditions in both cohorts relative to all DS individuals in the dataset were calculated and visualized using hierarchical clustering^44^. A cluster of co-occurring conditions with increased prevalences in *SOD1*-high but decreased in *SOD1*-low DS individuals (Figure 3B) was observed, including several co-occurring conditions associated with overactive or dysregulated immune cell function. A significantly higher odds ratio for pharyngitis in *SOD1*-high DS individuals versus *SOD1*-low was observed. (Figure 3C). Recurrent respiratory infections (i.e. pharyngitis) and increased risk of autoimmunity (i.e. psoriasis and seborrheic dermatitis) have been previously associated with decreased B and T lymphocyte viability in DS^4,7^. Our results demonstrate that *SOD1* overexpression may be associated with dysregulated immune cell function in DS, with adverse consequences to patient health.

Another cluster of co-occurring conditions with increased prevalences in the *SOD1*-low DS cohort, included the prevalence of acute myeloid leukemia (Figure 3B). Both low and high *SOD1* expression contribute to the elevated oxidative stress burden in DS. Low *SOD1* expression may contribute via decreased dismutation of excess superoxide radicals, and high *SOD1* expression via increased generation of hydrogen peroxide, a precursor to more damaging ROS such as the hydroxyl radical (HO^*−^)^14^. Our observations are entirely consistent with previous cellular studies linking elevated oxidative stress in DS to dysregulated hematopoiesis and an elevated risk for leukemia development^5,8^.

**Figure 4:**
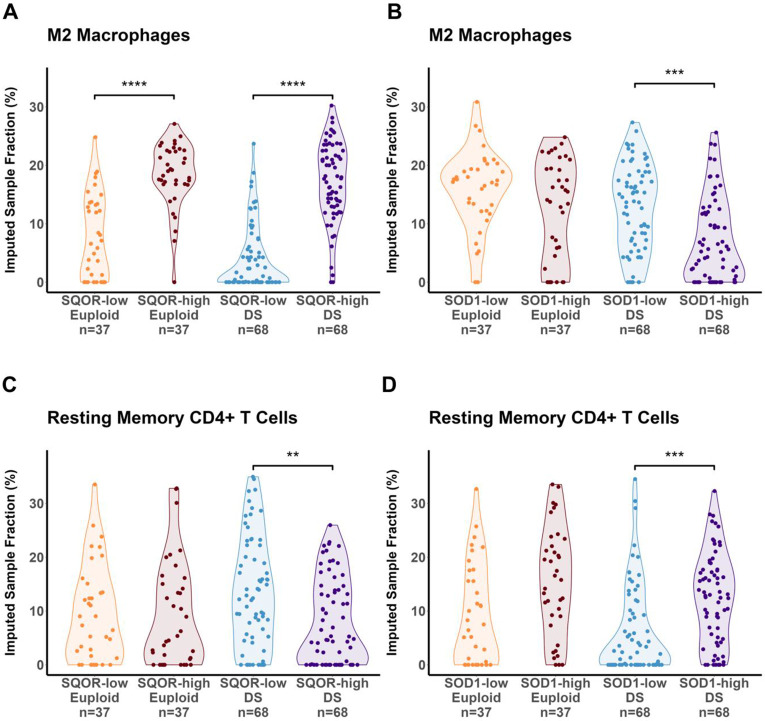
Divergent trends in inferred M2 macrophage and resting memory CD4+ T cell populations in *SQOR*- and *SOD1*-overexpressing DS individuals. Violin plots following deconvolution of bulk mRNA transcriptomic data from *SQOR*- and *SOD1*-high/low euploid and DS individuals revealed **A.)** increased M2 macrophage fractions in *SQOR*-high DS and euploid individuals relative to respective *SQOR*-low cohorts, yet **B.)** decreased M2 macrophage fractions in *SOD1*-high versus -low DS individuals but not in euploid, **C.)** decreased resting memory CD4+ T cell fractions in *SQOR*-high versus -low DS individuals but not in euploid, yet **D.)** increased resting memory CD4+ T cell fractions in *SOD1*-high versus -low individuals. These observations indicate that despite sharing a role as sulfide-oxidizing enzymes, *SQOR* overexpression is associated with resilience to inflammation while *SOD1* overexpression is linked with a pro-inflammatory state in DS. High and low expression cohorts defined as the 1^st^ and 4^th^ quartiles of *SQOR* and *SOD1* expression in euploid and DS separately. Dots indicate individuals. Kruskal-Wallis test, Dunn post-hoc. **:p < 1x10^−2^; ***:p < 1x10^−3^; ****:p < 1x10^−4^

Since *SOD1* also catalyzes the protective oxidation of H_2_S, we next compared the associations of high *SOD1* expression with the canonical H_2_S-oxidizing enzyme sulfide:quinone oxidoreductase (*SQOR*) and immune cell frequencies in DS. As above, high and low *SOD1* and *SQOR* expression were defined as the 1^st^ and 4^th^ quartiles respectively, in euploid and DS individuals. The bulk mRNA transcriptomic profile of individuals in each high- and low-expression cohort was deconvoluted using CIBERSORTx to infer immune cell subpopulation frequencies ^35^. In both euploid and DS, *SQOR*-high individuals had a significantly higher mean sample fraction of M2-polarized macrophages relative to respective *SQOR*-low (Figure 4A). Functionally different from pro-inflammatory M1 macrophages, M2-polarized macrophages are associated with wound healing and inflammation resolution ^45^. The greater M2-macrophage fraction in *SQOR*-high individuals is consistent with the idea that oxidized sulfur species increase M2 polarization, as shown previously by treatment with the long-acting H_2_S and polysulfide donor GYY4137. ^46-48 49^. It is possible that increased M2 polarization mitigates any pro-inflammatory macrophage activation in DS ^3^. As such, *SQOR* overexpression may be a potentially protective adaptation. In contrast, significantly lower M2 macrophage fraction in *SOD1*-high DS individuals was identified relative to *SOD1*-low DS (Figure 4B), suggesting a possible loss of resilience to a pro-inflammatory milieu with *SOD1* overexpression.

**Figure 5:**
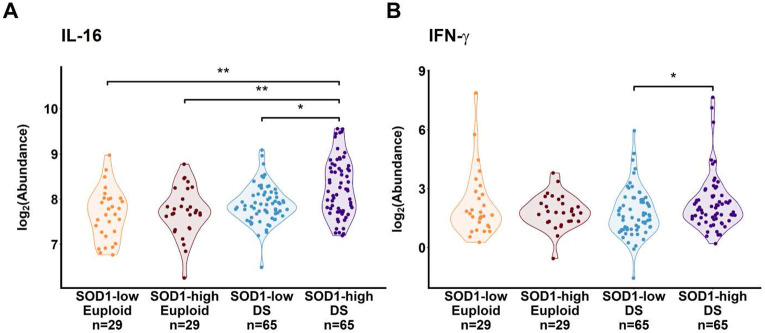
Elevated circulating levels of pro-inflammatory cytokines IL-16 and IFN-γ in *SOD1*-overexpressing DS individuals. Violin plots of interleukin-16 (IL-16) and interferon-γ (IFN-γ) in *SOD1*-high/low euploid and DS individuals showed **A.)** higher serum IL-16 levels in *SOD1*-high DS individuals relative to all other cohorts and **B.)** higher serum IFN-γ levels, indicating a pro-inflammatory paradigm closely linked with hyperactive CD4+ T cell activity. Y-axes denote log-normalized mass spectrometric abundances. Dots represent individuals. Kruskal-Wallis test, Dunn post-hoc. *:p < 5x10^−2^; **:p < 1x10^−2^

Furthermore, significantly lower resting memory CD4+ T cells were also identified in *SQOR*-high versus *SQOR*-low DS (but not euploid) cohorts (Figure 4C). In addition, a significantly higher memory CD4+ T cells fraction was also observed in *SOD1*-high versus *SOD1*-low DS individuals (Figure 4D). Serum proteomic analysis revealed significantly higher levels of pro-inflammatory cytokines such as interleukin-16 (IL-16) and interferon gamma (IFN-γ) in *SOD1*-high versus *SOD1*-low DS (Figure 5A-B). While memory CD4+ T cells are essential for the coordination of innate and adaptive immune responses in healthy individuals ^50^, lower stimulus threshold for activation relative to non-memory cells has implicated them as drivers of autoimmunity ^51^. IL-16 is a crucial chemoattractant and activator of CD4+ T cells, which release IFN-γ to polarize macrophages into the M1 paradigm, thereby potentiating inflammation^52,53^. Indeed, an increased frequency of memory CD4+ T cells and elevated levels of IL-16 and IFN-γ are recognized as components of a hyperactive inflammatory state and correspondingly high risk of autoimmunity in DS ^3,54^. A greater fraction of memory CD4+ T cells is consistent with observations of higher inflammatory disease risk in *SOD1*-high DS individuals, and merits future investigation using single-cell transcriptomics and *in vivo* DS mouse models.

**Figure 6:**
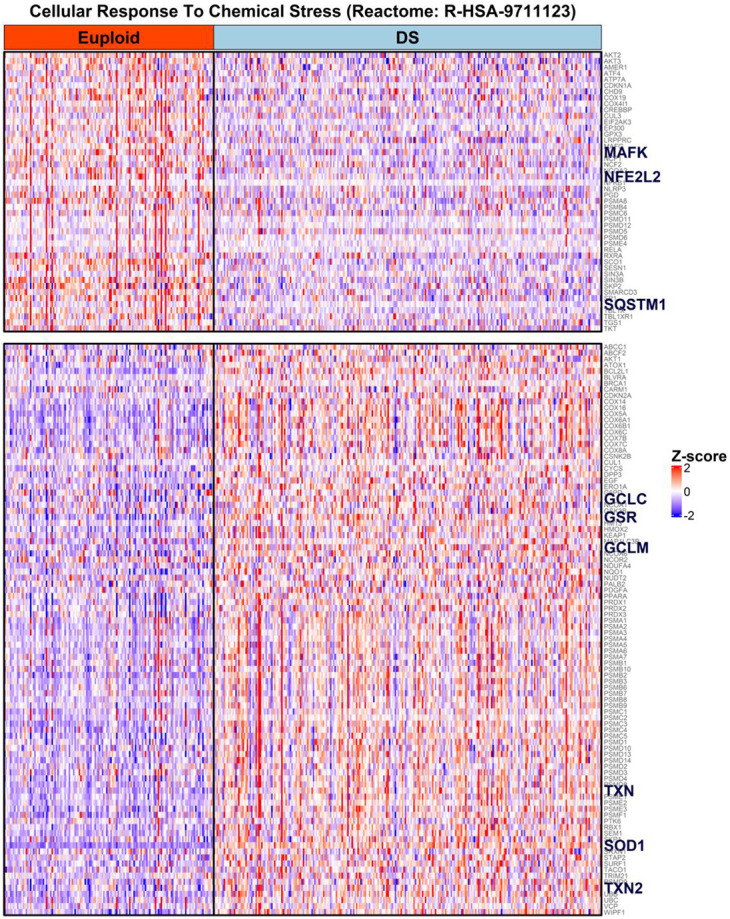
SOD1 co-overexpressed with oxidative stress response effectors. Overexpressed genes (lower group) include those involved in the synthesis (*GCLC*, *GCLM*) and regeneration (*GSR*) of reduced glutathione, thioredoxins (*TXN, TXN2*) and superoxide dismutase 1 (*SOD1*). A group of under-expressed oxidative stress response effectors (upper) was also observed, including Nrf2 (*NFE2L2*), a small Maf co-activator of Nrf2 (*MAFK*) and a regulator of mitophagy (*SQSTM1*), potentially indicating decreased resilience to oxidative stress in DS. Rows represent genes, columns represent euploid (red header) or DS (aqua header) individuals, with cell color indicating the row-wise Z-score of normalized mRNA count-per-million values across all DS and euploid individuals combined (red: overexpression; blue: underexpression, relative to all individuals in dataset).

Next, to understand whether *SOD1* overexpression is associated with altered patterns of DS gene expression biological pathways co-overexpressed with *SOD1* mRNA were examined using gene set enrichment analysis (GSEA). GSEA results were correlated with underlying biological mechanisms using active subnetworks of connections between biologically related genes. Individual genes that were significantly over- or under-expressed in DS were identified using the R pathfindR library, with protein-protein interaction information supplied from the Search Tool for the Retrieval of Interacting Genes/Proteins (STRING) database^33,55^. Subsequently, GSEA was performed on identified gene subnetworks and results mapped onto Reactome biological pathway gene sets^56^, identifying 484 significantly over- and underexpressed gene pathways in DS. Of the 484 pathway gene sets, only 1 pathway included *SOD1* (“Cellular Response to Chemical Stress”, Reactome ID # R-HSA-9711123), which contains genes involved in the detoxification of reactive oxygen and nitrogen species (ROS and RNS, respectively). DS-overexpressed genes in this set included those involved in the synthesis (*GCLC*, *GCLM*) and regeneration (*GSR*) of reduced glutathione (GSH), an essential peptide co-reactant for ROS detoxification and the reversal of ROS-mediated injuries^25^ (Figure 6, 7A-C). The co-overexpression of *SOD1* with genes involved in glutathione and thioredoxin/peroxiredoxin-mediated reversal of intracellular oxidative damage in DS suggests that oxidative consequences of *SOD1* overexpression are linked with a reflexive cellular drive to mitigate ROS-mediated damage.

Also overexpressed in DS were thioredoxins (*TXN, TXN2*) and thioredoxin-dependent peroxiredoxins (*PRDX1*, *PRDX2*, *PRDX3*). Peroxiredoxins are essential, cysteine-containing enzymes that reduce H_2_O_2_ and organic (i.e. lipid, DNA) peroxides^16^, levels of which are pathologically elevated in cells from DS mouse^15^ and human models^13,18,57^. *PRDX2* is the dominantly expressed cytosolic peroxiredoxin in WBCs and neurons ^58^ and was recently shown elevated in DS iPSC-derived neural progenitor cells ^59^. We report a significant positive correlation between *SOD1* and *PRDX2* mRNA expression in euploid and DS WBCs (Supplementary Figure 2). Also, *SOD1/PRDX2* mRNA expression ratios were 23.5% higher in the DS cohort relative to euploid (Supplementary Figure 3). These findings suggest that DS WBCs exhibit excess *SOD1*-mediated H_2_O_2_ generation relative to cytosolic decomposition versus euploid cells, and that the overexpression of antioxidant effectors may not be sufficient to counteract ROS generation in DS.

Intriguingly, a subset of genes within the same “Cellular Response to Chemical Stress” gene set were significantly underexpressed in DS relative to euploid (*NFE2L2*, *MAFK* and *SQSTM1*) (Figure 6). *NFE2L2* encodes the nuclear factor erythroid 2-related factor 2 (Nrf2) transcription factor which is co-activated by transcription factors of the small Maf family (i.e. *MAFK)* to stimulate the expression of antioxidative response genes^60^. Underexpression of *NFE2L2* and *MAFK* mRNA in DS may therefore suggest an impaired capacity to activate Nrf2-regulated oxidative damage response effectors, potentially indicating decreased resilience to oxidative stress. Furthermore, the under expression of the mitophagy regulator sequestosome 1 (*SQSTM1*) has been linked to impaired mitophagy and accumulation of damaged mitochondria in DS cells, which exacerbates the oxidative stress burden via increased superoxide generation^61^. Our results are consistent with prior experimental data indicating decreased resilience to, and increased generation of ROS in DS cells ^14,61^ and support compensatory reliance on intracellular thiol antioxidant (glutathione and thioredoxin-mediated) systems.

**Figure 8:**
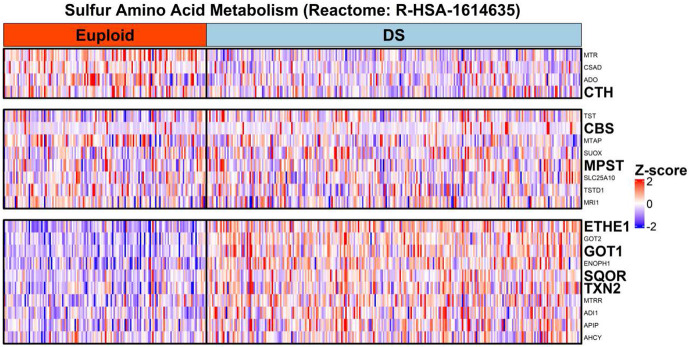
Overexpression of genes participating in sulfur amino acid and hydrogen sulfide metabolism in DS WBCs. mRNA expression analysis of genes involved in sulfur amino acid metabolism in euploid and DS WBCs reveals three groups: genes underexpressed (upper), overexpressed (lower) and similarly expressed (middle) in DS relative to euploid WBCs. Notably, components of the *CBS* and *CTH*-catalyzed reverse transsulfuration pathway were observed to be similarly or underexpressed respectively in DS, suggesting a lesser contributory role toward H_2_S generation. While *MPST* mRNA was also found to be similar across DS and euploid individuals, components of the cysteine catabolic pathway essential to *MPST*-catalyzed H_2_S generation – including cysteine aminotransferases (*GOT1*, *GOT2*) and thioredoxins (*TXN2*) – were overexpressed in DS. Furthermore, the expression of enzymes involved in H_2_S and polysulfide oxidation (*SQOR*, *ETHE1*) were also elevated across DS individuals relative to euploid. Together, these results support a stronger role of *MPST* in H_2_S overproduction and an increased drive toward H_2_S detoxification in DS WBCs. Rows represent genes, columns represent euploid (red header) or DS (aqua header) individuals, with cell color indicating the row-wise Z-score of normalized mRNA count-per-million values across all DS and euploid individuals combined (red: overexpression; blue: underexpression, relative to all individuals in dataset).

The observation that *SOD1* co-overexpression with thiol antioxidant system effectors has important implications for hydrogen sulfide (H_2_S) generation in DS cells. In addition to their essential role as co-reactants in cellular antioxidant pathways, glutathione and thioredoxin are important components of intracellular H_2_S detoxification and generation pathways respectively ^26,49^. H_2_S overproduction is a recognized feature of DS molecular pathophysiology ^19,62^ and is associated with decreased oxidative phosphorylation ^18,19^. Endogenous H_2_S generation can occur through cystathionine-β-synthase (*CBS*)- and cystathionine-γ-lyase (*CTH*)-dependent cysteine synthesis from homocysteine (reverse transsulfuration) and cysteine catabolism via 3-mercaptopyruvate sulfurtransferase (*MPST*)^26^. In the latter pathway, *MPST* reacts with the cysteine transamination catabolite 3-mercaptopyruvate, generating a persulfidated enzyme intermediate that can react with thioredoxins to liberate H_2_S^26,63^. Heatmap analysis of genes participating in sulfur amino acid metabolism (Reactome ID #R-HSA-1614635) indicated the overexpression of key genes related to thioredoxin/MPST-mediated H_2_S generation, including thioredoxin 2 (*TXN2*) and cytosolic (*GOT1*) as well as mitochondrial aspartate aminotransferase (*GOT2*), the latter 2 enzymes being the principal source of intracellular 3-mercaptopyruvate generation via cysteine transamination^26,64^ (Figure 8, 9A-B). In contrast, *CTH* mRNA was significantly under-expressed in DS individuals relative to euploid, and no significant difference in *CBS* expression was detected between DS and euploid. These results suggest that a cysteine transamination, MPST and thioredoxin-dependent axis drives the generation of excess H_2_S in DS WBCs.

In addition to the *CBS*- and *MPST*-catalyzed routes, H_2_S can also be generated by cysteinyl-tRNA synthetases, which were not included in the Reactome list of genes participating in sulfur amino acid metabolism at the time of our analysis. While the expression of the cytosolic cysteinyl-tRNA synthetase 1 (*CARS1*) was not different between the two cohorts, the expression of the mitochondrial *CARS2* was significantly lower in DS (Supplementary Figure 4). Together, these findings support a decreased role of *CARS1/2*-catalyzed generation toward H_2_S overproduction in DS WBCs.

**Figure 10:**
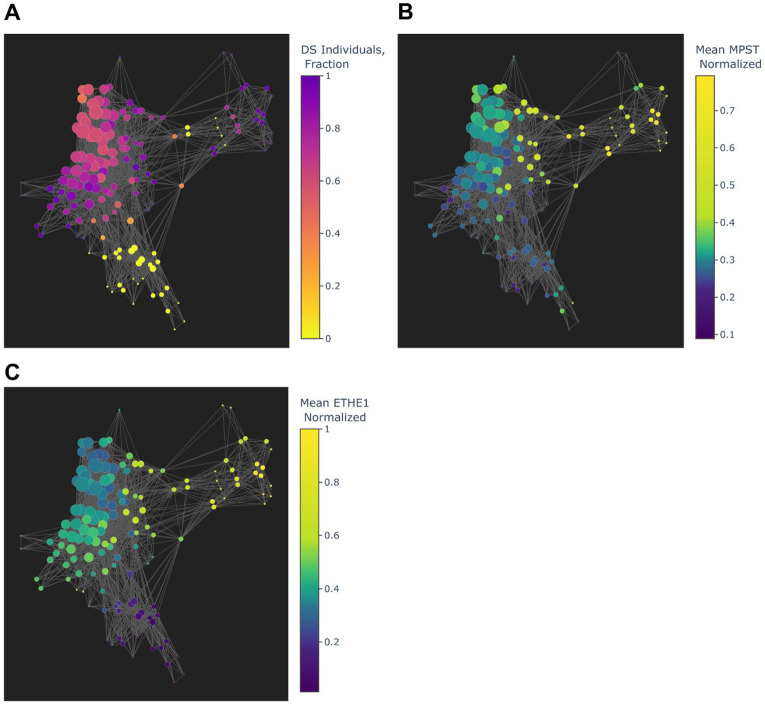
Topological representation of mRNA profiles distinguishes euploid from DS individuals based on cysteine transamination and sulfur metabolic genes, excluding *CBS*. Graphs generated using an Isolation Forest anomaly score calculated from the expression of non-chromosome 21 genes involved in cysteine catabolism-linked H_2_S generation – *MPST*, *SQOR*, *ETHE1*, *TXN2*, *TST*, *CTH*, *GOT1*, *GOT2* - and Principal Component Analysis as filter functions for the Mapper algorithm. Nodes represent groups of individuals with similar mRNA expression profiles and edges represent similarities between nodes. **A.)** Coloring of nodes by fraction of DS individuals (“1” represents 100% of individuals in a node being from the DS cohort) reveals that expression of cysteine catabolism-associated genes separates DS-dominant nodes from euploid **B.)** Coloring of nodes by mean *MPST* expression (“1” represents highest mRNA expression) indicates higher *MPST* expression levels in DS-dominant nodes, concurrent with **C)** higher *ETHE1* expression in DS-dominant nodes. Elevated *MPST* and *ETHE1* suggest increased cysteine catabolism-linked H_2_S generation and oxidation respectively in DS cells.

**Figure 11:**
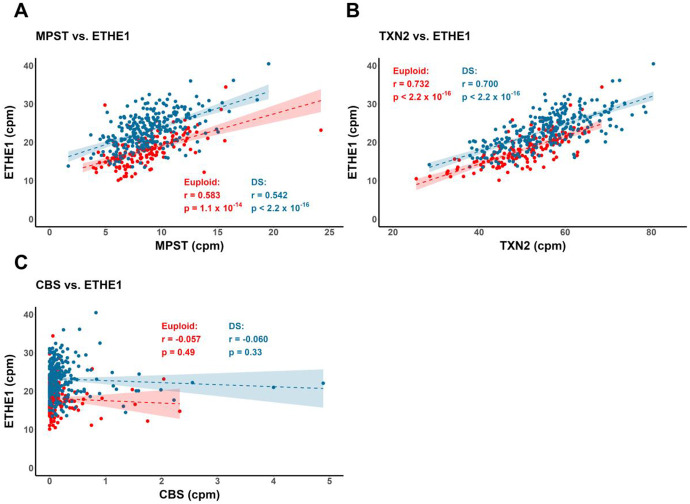
Correlations of mRNA expression between H_2_S generation and oxidation pathways. Significant positive correlations between **A.)** *MPST* (H_2_S generation) versus *ETHE1* (H_2_S oxidation) and **B.)** *TXN2* versus *ETHE1* mRNA expression across euploid and DS WBCs, separately. **C.)** No significant correlation between *CBS* and *ETHE1* observed, supporting a smaller role of CBS-mediated H_2_S production in WBCs. Gene expression values reported as normalized counts-per-million, with dots representing individuals (euploid n = 146; DS n = 270). Pearson’s *r*.

Overexpression of sulfide:quinone oxidoreductase (*SQOR*) and persulfide dioxygenase (*ETHE1*) was also observed in DS versus euploid (Figure 8, 9C-D). *SQOR* and *ETHE1* catalyze the canonical, glutathione-dependent detoxification of H_2_S to polysulfides and sulfite (SO_3_^2−^) respectively, and their overexpression suggests a greater intracellular demand for H_2_S detoxification in DS^49^. To characterize the association, if any, between cysteine catabolism-linked H_2_S generation and H_2_S oxidation pathways in the context of DS, we used topological data analysis to visualize group dissimilarities in gene expression by calculating an anomaly score using the mRNA expression levels of *MPST*, *SQOR*, *ETHE1*, *TXN2*, *TST*, *CTH*, *GOT1* and *GOT2*. We observed a notable separation between DS- and euploid-predominant nodes (Figure 10A), indicating that the expression of non-*Hsa21* genes linked to sulfur metabolism can distinguish clusters of DS individuals from euploid independent of *CBS* expression. The concurrent overexpression of *MPST* (Figure 10B) and *ETHE1* (Figure 10C) in DS-dominant nodes was also observed, suggesting elevated cysteine catabolism-linked H_2_S generation and sulfide oxidation. Additionally, significant positive correlations between *MPST*, *TXN2* and *ETHE1* mRNA – but not with *CBS* – in DS and euploid WBCs were observed, potentially indicating a stronger contributing role of an MPST-mediated H_2_S generation pathway to the sulfide burden in DS cells (Figure 11A-C).

## Discussion:

In this report, a novel transcriptomic and proteomic characterization uncovered an increased glutathione- and thioredoxin-dependent intracellular drive correlated with *SOD1* overexpression and H_2_S overproduction in DS WBCs. An important limitation of transcriptomic investigations is that despite highlighting networks of gene regulation, mRNA levels do not imply protein levels inside cells. Similarly, serum proteomics does not convey information regarding the tissue-specific distribution and dynamics of inflammation in DS. Furthermore, our analyses of co-occurring clinical conditions are limited by variability in the presentation and reporting of clinical symptoms. Despite these limitations, however,our observations are consistent with a cellular response to reverse oxidative stress-mediated injuries, previously related empirically elevated ROS production and catabolismin multiple cell and animal models of DS^6,8,12,14^. Increased imputed fractions of pro-inflammatory CD4+ T cells and the inflammation-associated cytokines IL-16 and IFN-γ with *SOD1* overexpression further implicate it as a contributor to immune dysregulation in DS^3^. Furthermore, the pro-inflammatory effects of *SOD1* overexpression contrast with the anti-inflammatory M2 macrophage polarization with that of *SQOR*. Despite the protective H_2_S oxidizing function, excess *SOD1* may negatively alter immune cell homeostasis in DS – a role that merits further investigation.

Others have identified mechanisms leading to the generation of excess oxidative stress in DS cells, such as *SOD1* triplication^12,65^, altered proteostasis ^66^ and dysregulated clearance of damaged mitochondria^61^. However, the underlying mechanism(s) supporting resilience to oxidative damage in DS cells remain unclear. Prior clinical studies have shown increased activities of glutathione peroxidases in DS erythrocytes^67,68^ and protein levels of peroxiredoxins 1 and 2 in specific brain regions from DS patients ^69^.

Peroxiredoxins and glutathione peroxidases are enzyme families that reduce the inorganic (H_2_O_2_) and organic peroxides that arise due to ROS-mediated damage ^16^. Both classes depend on thiol-containing peptide co-reactants, thioredoxins and glutathione respectively, as essential reducing agents. Thioredoxins are a family of proteins (~12 kDa) that contain a conserved cysteine-glycine-proline-cysteine (CGPC) motif, allowing intramolecular disulfide bonds to form upon oxidation and constituting an integral aspect of their antioxidant function ^70^. Our findings of increased thioredoxin (*TXN*, *TXN2*) gene expression in DS WBCs suggest an increased intracellular demand for thiol antioxidant co-reactants to reverse oxidative damage. Increased transcription also suggests that normal NADPH-dependent regeneration of thioredoxins ^70^ is insufficient to meet heightened demand in DS cells. This idea is consistent with prior studies showing that DS neurons are more susceptible to cell death following inhibition of thioredoxin redox cycling with auranofin and indicate an increased sensitivity to thiol antioxidant depletion ^71^.

In addition to the overexpression of thioredoxins, our analyses uncover evidence for the increased regeneration and *de novo* synthesis of glutathione in DS. Glutathione is a tripeptide composed of glutamate, cysteine and glycine and is the most abundant endogenous antioxidant in humans^72,73^. As a co-reactant for antioxidant enzymes, reduced glutathione (GSH) donates electrons toward the reduction of organic peroxides, forming oxidized glutathione (GSSG). GSSG is a disulfide derived from two GSH molecules and can be reduced to regenerate GSH upon NADPH-dependent reduction in a reaction catalyzed by the enzyme glutathione reductase (GSR) ^25^. GSH can also be synthesized *de novo*, and the rate limiting step of GSH synthesis is the conjugation of cysteine to glutamate catalyzed by glutamate cysteine ligase (GCL), encoded by the *GCLC* and *GCLM* genes^73^.

The increased *GSR* gene expression reported here is a potential mechanism to explain reports of higher GSR activities in whole blood from DS individuals relative to euploid^65^, suggesting accelerated glutathione turnover due to increased oxidation. Garlet et al. ^65^ also reported that serum GSH levels were lower in DS individuals relative to euploid^65^. As such, a combination of decreased blood GSH together with elevated glutathione peroxidase activity may be indicative of an increased reliance on GSH-dependent antioxidant pathways leading to accelerated GSH depletion. Increased *de novo* synthesis of GSH - supported by *GCLC* and *GCLM* overexpression– provides a compensatory mechanism to replenish GSH stores within DS cells. Furthermore, *GCLC* and *GCLM* overexpression is concordant with increased serum cysteine levels in DS individuals relative to euploid controls ^74^, indicating increased cysteine import linked to *de novo* GSH synthesis. Our findings motivate further investigations into the post-transcriptional regulation of GCL activity and its contribution to inadequate matching of GSH supply and demand in DS cells.

In addition to its role as an essential antioxidant, GSH is also a co-reactant in mitochondrial sulfur oxidation pathways, an essential mechanism for intracellular H_2_S detoxification ^26,49,75^. In the presence of GSH, sulfide:quinone oxidoreductase (*SQOR*) acts in mitochondria to oxidize hydrogen sulfide ^49^ *SQOR* facilitates the transfer of sulfur atoms from H_2_S to form glutathione persulfides (GSSH), which are oxidized by *ETHE1* (persulfide dioxygenase) and *TST* (thiosulfate sulfurtransferase) to yield sulfite and thiosulfate oxidation products respectively^49^. It is possible that H_2_S overproduction in DS contributes to increased GSH production as part of an increased need for H_2_S detoxification, an idea supported by the present findings of increased *SQOR* and *ETHE1* gene expression in DS WBCs. An important caveat is that GSH and thioredoxin-dependent antioxidant effectors can also metabolize H_2_S and other reactive sulfur species (RSS) ^76^. The overlapping cellular responses to RSS and ROS make it difficult to distinguish the effects of one versus the other on the WBC transcriptome. Future experiments are therefore needed to determine the effects of glutathione and cysteine metabolism in DS on cellular responses to ROS and RSS.

**Figure 12:**
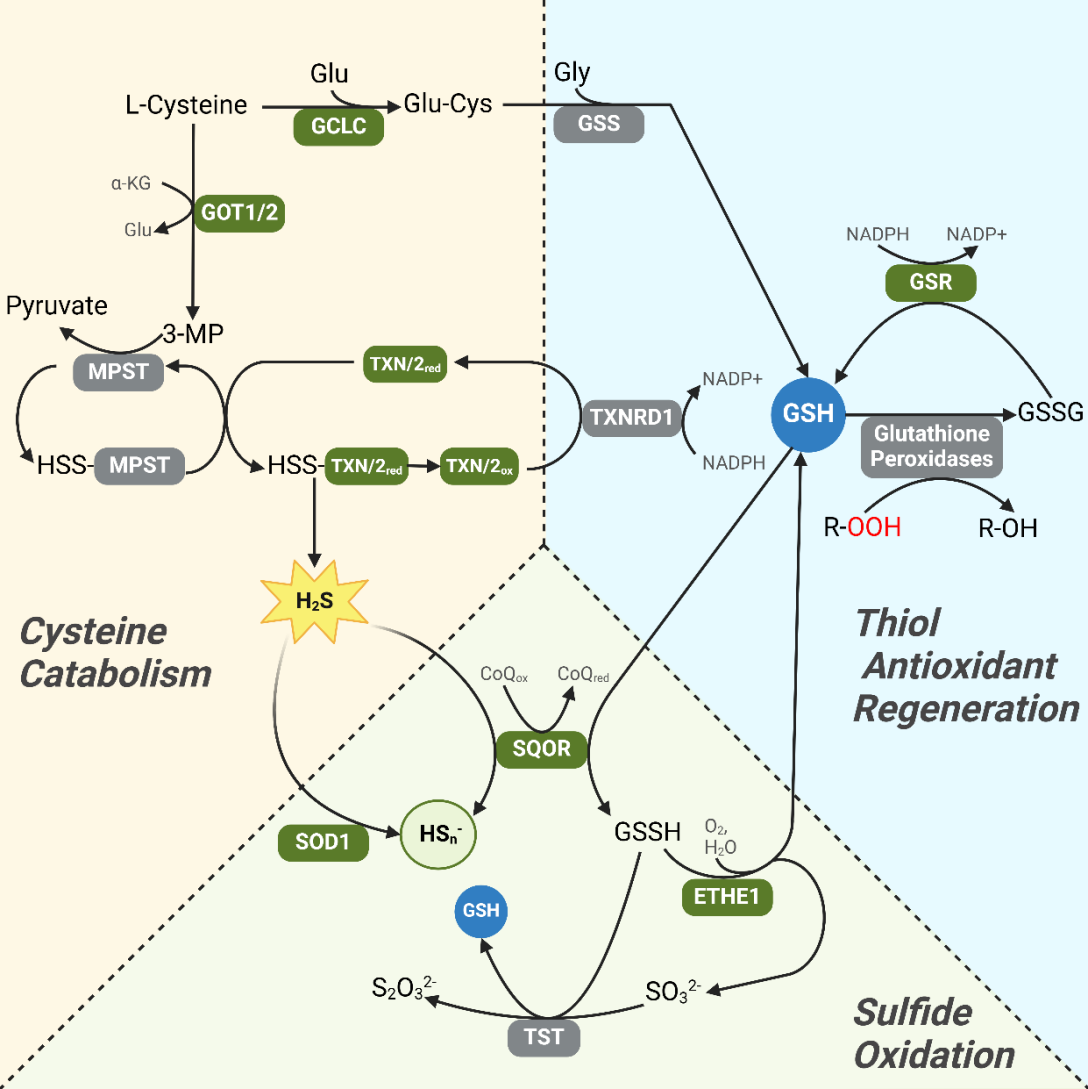
Pathways of cysteine catabolism, sulfide oxidation and the regeneration of the endogenous thiol antioxidants glutathione (GSH) and thioredoxins (TXN/2) are intimately linked and overexpressed in DS. Green symbols represent genes transcriptionally overexpressed in DS WBCs. Reduced glutathione (GSH) is indicated in blue, and peroxidation (−OOH) is indicated in red. Cysteine is transaminated by aspartate aminotransferase (GOT1/2), generating 3-mercaptopyruvate (3-MP). The thiol group of 3-MP is transferred as a persulfide to 3-mercaptopyurvate sulfurtransferase (*MPST*), and released as H_2_S upon reaction with thioredoxins. mRNA overexpression of *GOT1/2* and *TXN/2* may thus drive H_2_S overproduction via accelerated cysteine catabolism in DS cells. Removal of cytotoxic H_2_S from cells depends on GSH-mediated oxidation by sulfide:quinone oxidoreductase (*SQOR*), generating glutathione polysulfides (GSSH) which are converted to GSH by persulfide dioxygenase (*ETHE1*), generating sulfite (SO_3_^2−^) and thiosulfate anions (S_2_O_3_^2−^) after further reaction with thiosulfate sulfurtransferase (*TST*). H_2_S is also directly oxidized to polysulfides (HS_n_^−^) by *SOD1*, representing an alternative avenue of protective sulfide oxidation. Overexpression of *SQOR*, *ETHE1* and *SOD1* may indicate an increased drive toward sulfide oxidation due to a cytotoxic H_2_S excess in DS cells. GSH synthesis and regeneration is also linked with cysteine metabolism through the *de novo* synthetic pathway catalyzed by glutamine-cysteine ligase (*GCLC*) and glutathione synthetase (*GSS*). GSH is an essential co-reactant for glutathione peroxidase-mediated reversal of lipid and protein peroxidation (R-OOH), a toxic consequence of reactive oxygen species (ROS). Overexpression of glutathione reductase (*GSR*) - an enzyme which regenerates GSH from oxidized (GSSG) glutathione – suggests a cellular response to increased ROS-mediated damage in DS cells, indicating concurrent oxidative and sulfide-mediated stress. Other abbreviations: α-KG: α-ketoglutarate; Glu: glutamine; Gly: glycine; CoQ_ox_, CoQ_red_: oxidized and reduced coenzyme Q

Together with increased thiol antioxidant metabolism and regeneration, we report a correlation between increased cysteine catabolism and H_2_S generation in DS WBCs (Figure 12), but not with *CBS*. A lack of correlation between H_2_S overproduction and *CBS* levels is supported by our report of elevated H_2_S and polysulfide levels in DS B lymphocytes relative to euploid cells, despite no significant differences in CBS protein levels. ^18^. Differences in cell lineage may account for the notable variability in CBS overexpression across multiple systems in humans and animal models^19^ and thus, future experiments utilizing single-cell RNA sequencing to identify the effect of cell type on CBS expression will be useful. As mRNA expression and protein levels may not reflect protein activity, additional experiments are needed to evaluate post-translational and metabolic control of *CBS* activity in DS WBCs to better assess the contribution to H_2_S overproduction.

In contrast, our findings of increased aminotransferase and thioredoxin expression implicate *MPST* as a potential driver of H_2_S overproduction in DS WBCs. *MPST* catalyzes the decomposition of 3-mercaptopyruvate to pyruvate and H_2_S in the presence of thiol-containing species such as lipoic acid, cysteine and thioredoxins^26^. 3-mercaptopyruvate, in turn, is generated from the transamination of cysteine by aspartate and glutamate aminotransferases (*GOT1/2*)^75^. *GOT1/2* and *TXN* overexpression in DS. Therefore, these data suggest an enhanced capacity for *MPST*-mediated H_2_S generation through the excess generation and decomposition of 3-mercaptopyruvate, respectively. While a prior study by Panagaki et al. reported higher levels of *MPST* in DS dermal fibroblasts relative to euploid controls^24^, we did not observe significantly elevated *MPST* mRNA expression in DS WBCs^26,75^. It must be noted that *MPST*-mediated H_2_S generation is sensitive to the concentration of specific sulfide acceptors and increases together with thioredoxin concentrations at physiological levels ^77^. Increased thioredoxin expression in DS cells likely synergizes with *MPST* activity to drive H_2_S overproduction in the oxidatively stressed DS cell. ^77^

Our findings therefore suggest that H_2_S overproduction is a response to the oxidative consequences of *Hsa21* trisomy. One such consequence is increased per-mitochondrion superoxide generation, previously attributed to dysregulated mitochondrial biogenesis and autophagy dynamics ^14,61,78^. As H_2_S directly reacts with superoxide ^79^, reflexive upregulation of H_2_S production may serve as a protective buffer against increased trisomy 21-related mitochondrial ROS generation. H_2_S also serves as the precursor for polysulfides, which readily react with superoxide ^62,80^ and modify cysteine residues by persulfidation. Cysteine persulfidation is protective modification which shields its side chain sulfur from irreversible ROS-mediated oxidation, thus preserving its essential catalytic function in various proteins ^81^. Particularly, the persulfidation of active site cysteines in *PRDX2* protect it against H_2_O_2_-mediated overoxidation, which would otherwise lead to the formation of catalytically inactive aggregates ^58,82^. A larger “polysulfide pool” because of increased H_2_S generation may thus promote cellular resilience to ROS in DS, meriting further study. Despite its potential protective effects, it is still possible that H_2_S overproduction represents a “metabolic overcorrection” to increased ROS generation in DS, and future investigations are needed to determine the mechanisms that sustain overproduction in the absence of *CBS* overproduction.

In summary, we report a series of novel mechanisms underlying a thiol antioxidant axis-driven response to oxidative stress in DS WBCs that provides a possible explanation for H_2_S overproduction. Future studies targeting the crosstalk between ROS and H_2_S metabolism are warranted to improve WBC health and their effects on immune function in DS.

## Supplementary Material

Supplement 1

## Figures and Tables

**Figure 7:**
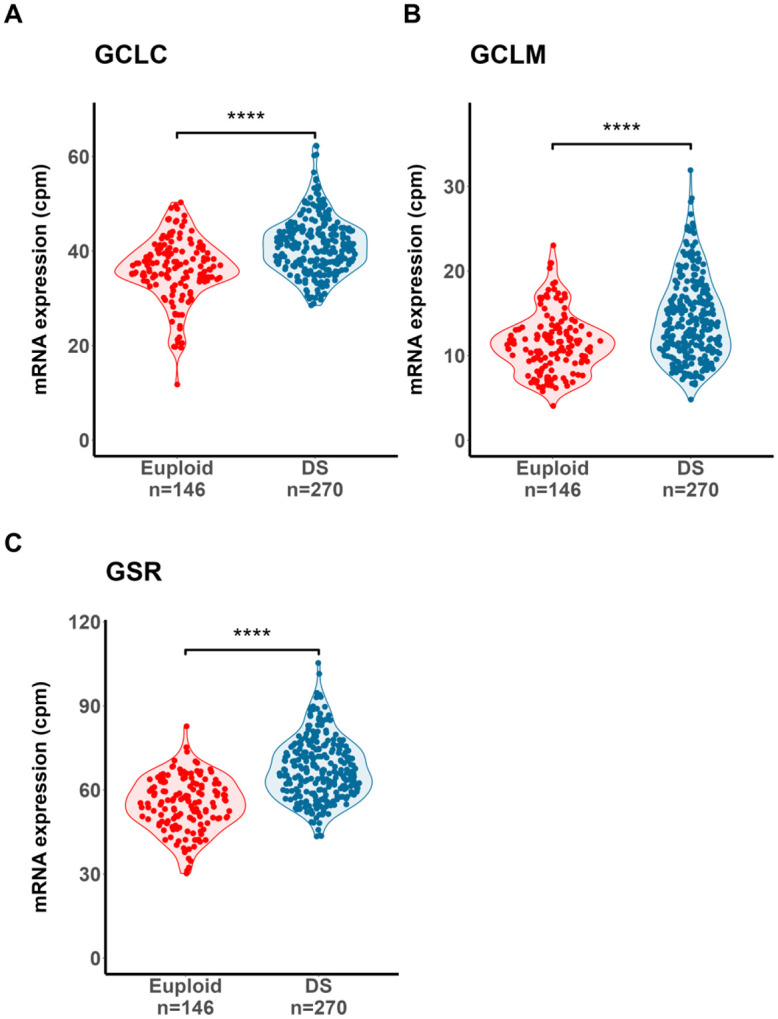
Overexpression of genes involved in glutathione metabolism in DS WBCs. Violin plots highlight significantly higher expression of **A.)** glutathione synthase catalytic (*GCLC*) and **B.)** regulatory subunits (*GCLM*) as well as **C.)** glutathione reductase (*GSR*) in DS individuals relative to euploid, supporting increased thiol antioxidant demand in response to oxidative stress. Y-axis indicates mRNA expression, measured as counts-per-million (cpm) following normalization. Dots denote individuals. P-values calculated from comparisons between DS and euploid individuals through likelihood-ratio tests using edgeR and adjusted for multiple comparisons using the Benjamini-Hochberg correction to obtain q-values. ****: q < 1x10^−8^.

**Figure 9:**
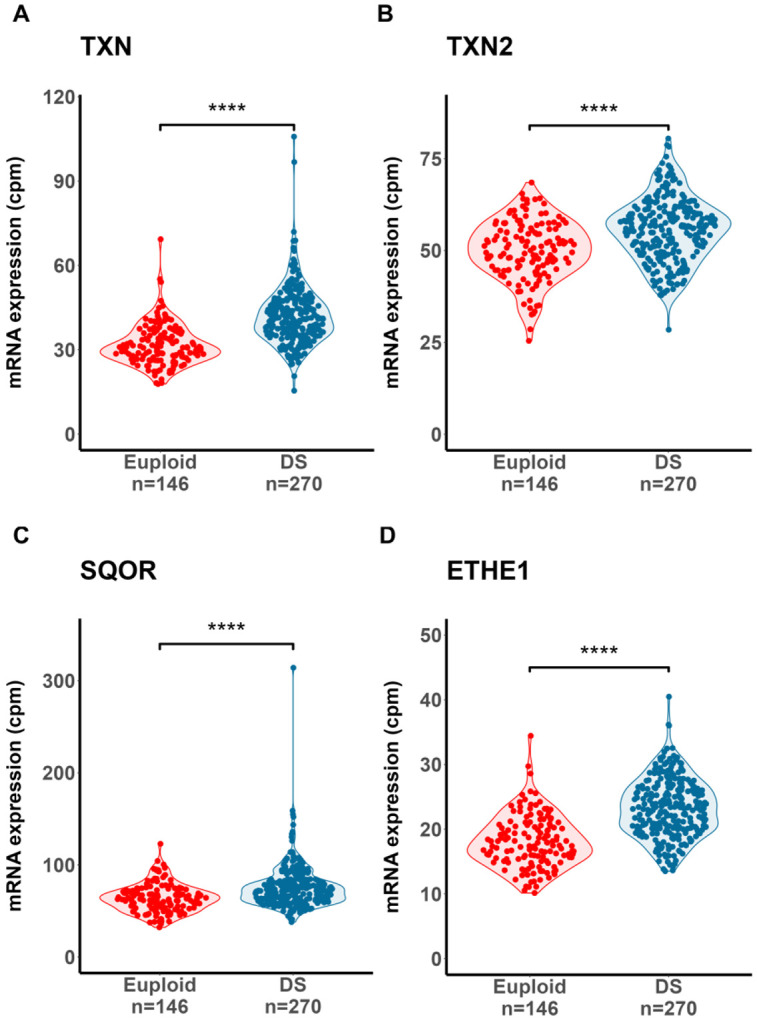
Overexpression of thioredoxins and key participants of hydrogen sulfide catabolism in DS. Violin plots indicate significantly higher expression of **A.)** thioredoxin (*TXN*) and **B.)** thioredoxin 2 (*TXN2*) in DS - necessary reducing agents in *MPST*/thioredoxin-mediated H_2_S generation. Elevated expression of the sulfide catabolism enzymes **C.)** sulfide:quinone oxidoreductase (*SQOR*) and **D.)** persulfide dioxygenase (*ETHE1*) supports the existence of a cellular response to excess H_2_S in DS WBCs. Y-axis indicates mRNA expression, measured as counts-per-million (cpm) following normalization. Dots denote individuals. P-values calculated from comparisons between DS and euploid individuals through likelihood-ratio tests using edgeR and adjusted for multiple comparisons using the Benjamini-Hochberg correction to obtain q-values. ****: q < 1x10^−8^.
