# Supplementary material for: SOD1 at the Crossroads: Co-Overexpression of Canonical Antioxidant Response and Noncanonical Hydrogen Sulfide Generation Pathways in Down Syndrome, With Immune Cell Implications
Source: Res Sq. 2026 Jan 13:rs.3.rs-8535243. Preprint. [Version 1] doi: 10.21203/rs.3.rs-8535243/v1 (PMC12869641; doi:10.21203/rs.3.rs-8535243/v1)
Supplement: Supplement 1 [file NIHPPrs8535243v1-supplement-1.pdf]

Supplementary Figures:

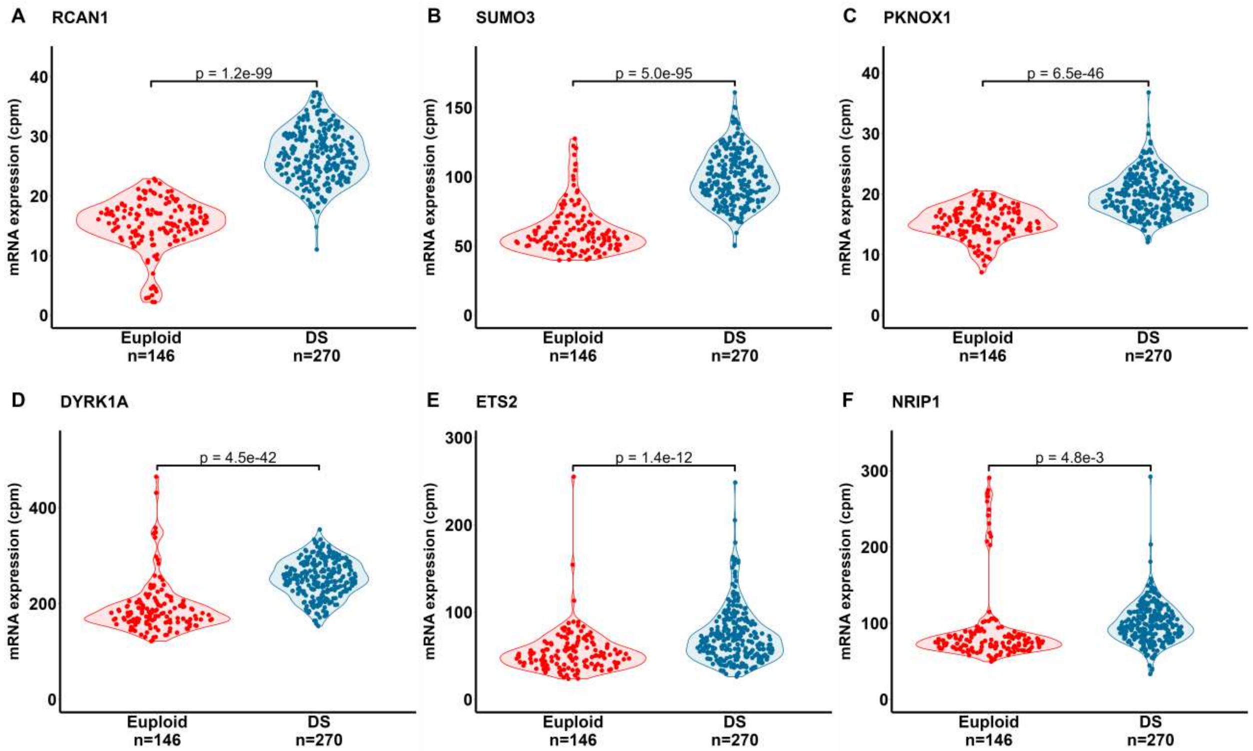

Supplementary Figure 1: Increased expression of Ch. 21 genes for which a dosage imbalance is implicated in dysregulated mitochondrial biogenesis and function [38,39]including a.) *RCAN1*, b.) *SUMO3*, c.) *PKNOX1/PREP1*, d.) *DYRK1A*, e.) *ETS2*, f.) *NRIP1*. Likelihood-ratio test, edgeR.

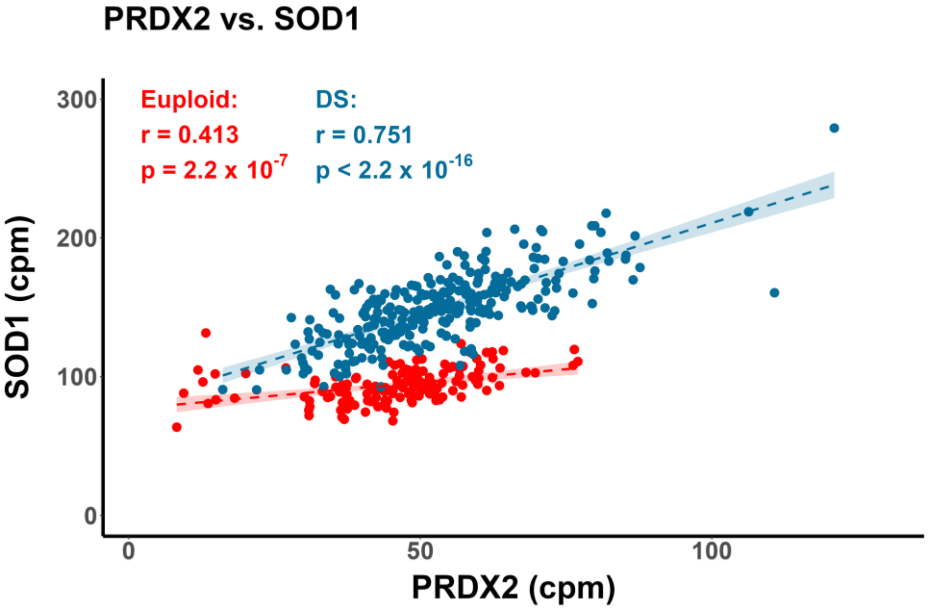

Supplementary Figure 2: *SOD1* and *PRDX2* positively correlated in euploid and DS individuals, represented by dots (n= 146 and 270 respectively). Pearson's r, linear regression t-test.

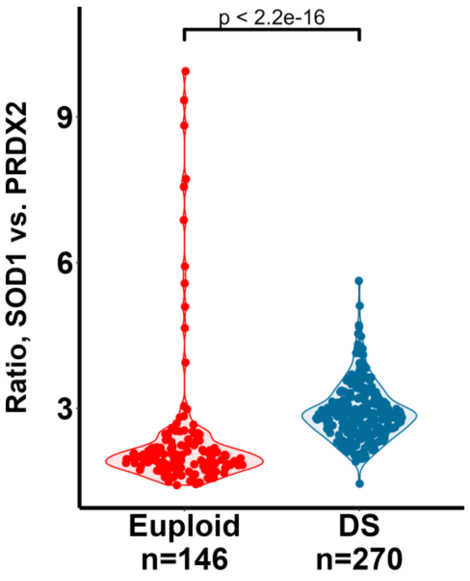

Supplementary Figure 3: Higher *SOD1/PRDX2* mRNA expression ratios in DS individuals relative to euploid. Each dot represents 1 individual. Mann-Whitney U-Test.

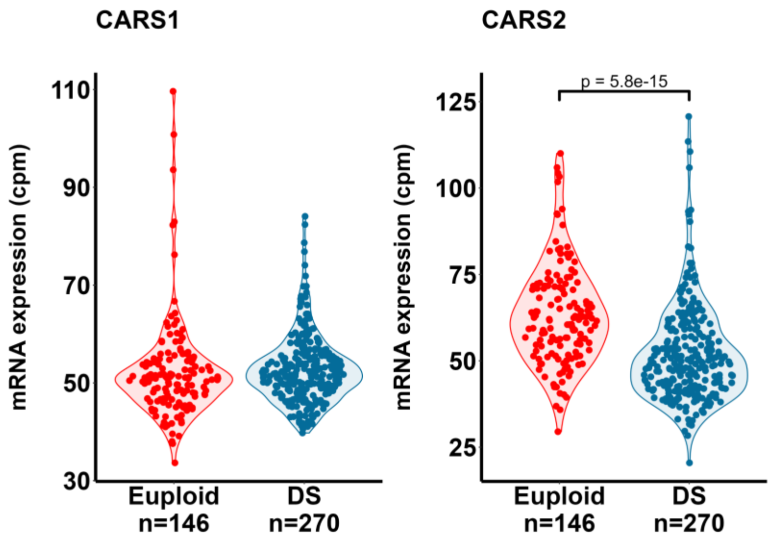

Supplementary Figure 4: Similar *CARS1* and lower *CARS2* mRNA expression in DS individuals relative to euploid. Each dot represents 1 individual. Likelihood-ratio test, *edgeR*.

2

5
